# Hemin inhibits NO production by IL-1β-stimulated human astrocytes through induction of heme oxygenase-1 and reduction of p38 MAPK activation

**DOI:** 10.1186/1742-2094-7-51

**Published:** 2010-09-07

**Authors:** Wen S Sheng, Shuxian Hu, Adam R Nettles, James R Lokensgard, Gregory M Vercellotti, R Bryan Rock

**Affiliations:** 1 The Center for Infectious Diseases & Microbiology Translational Research, Department of Medicine, University of Minnesota Medical School, Minneapolis, Minnesota, USA; 2 Division of Hematology, Oncology, and Transplantation, Department of Medicine, University of Minnesota Medical School, Minneapolis, Minnesota, USA

## Abstract

**Background:**

Heme oxygenase (HO)-1 has been shown to attenuate oxidative injury and reduce apoptosis. HO-1 can be induced by various stimuli released during cellular injury, such as heme. Deleterious free heme is degraded by HO-1 to carbon monoxide, iron and biliverdin, which have potent anti-oxidant and anti-inflammatory properties. In this study, we tested the hypothesis that upregulation of HO-1 would inhibit production of the free radical (NO) by interlukin (IL)-1β-activated human astrocytes.

**Methods:**

To measure NO production, inducible NO synthase (iNOS), HO-1 expression and mitogen-activated protein (MAP) kinase activation we used hemin as an HO-1 inducer and tin protoporphyrin (SnPP) IX as an inhibitor of HO-1 activity in human astrocyte cultures prior to IL-1β exposure. Transfection of astrocyte cultures was performed using a pLEX expression vector carrying the human HO-1 sequence prior to IL-1β treatment. Supernatants of astrocyte cultures pretreated with inhibitors of p38 MAPK or MEK1/2 prior to IL-1β exposure were collected for NO assay.

**Results:**

IL-1β treatment of astrocytes alone induced undetectable amounts of HO-1 protein by western blot. However, HO-1 mRNA expression was modestly up-regulated in response to IL-1β stimulation. Pretreatment with hemin alone substantially induced both HO-1 mRNA and protein expression, and HO-1 mRNA expression was further enhanced when hemin was combined with IL-1β treatment. In contrast, IL-1β-induced iNOS mRNA expression and NO production were markedly inhibited by hemin treatment. When pretreated with SnPP, the inhibitory effect of hemin on IL-1β-induced NO production and iNOS expression was reversed, suggesting the involvement of HO-1. IL-1β-induced p38 MAPK activation, which is known to be required for NO production, was also down-regulated by hemin.

**Conclusion:**

These findings support the hypothesis that up-regulation of HO-1 in astrocytes is associated with down-regulation of iNOS expression and thereby NO production, an effect that involves the p38 MAPK signaling pathway, which suggests that this glial cell response could play an important protective role against oxidative stress in the brain.

## Background

Hemin (ferriprotoporphyrin IX chloride), the oxidized form of the heme moiety of hemoglobin and a constituent of many enzymes, is degraded by heme oxygenase (HO)-1, which in turn generates carbon monoxide (CO), iron and biliverdin. While CO and biliverdin each have cytoprotective and anti-inflammatory properties, iron is sequestered by ferritin to reduce free radical formation and later utilized to maintain iron homeostasis for gene regulation. Hemin has been reported to suppress human immunodeficiency virus (HIV)-1 infection of human monocytes through HO-1 induction [1], but has also been reported to induce necroptosis of murine cortical astrocytes [2] and oxidative injury to human neurons [3]. In a recent study hemin was used to induce HO-1 in humans [4].

Under conditions of oxidative stress, induction of HO-1 is evident, and its anti-oxidant properties are thought to contribute to balancing the redox environment. HO-1, which is the inducible isoform of the stress response protein HO that detoxifies heme, can be induced by many stimuli and is considered a therapeutic funnel because its activity appears to be required by other therapeutic molecules [5]. Induction of HO-1 expression within the central nervous system (CNS) has been demonstrated in rodent astrocytes, microglia/macrophages and neurons [6-8]. However, neurons constitutively express primarily HO-2 under normal conditions [9] and rodent astrocytes also have been shown to express HO-2 [10]. Clinically, up-regulated HO-1 expression appears to be beneficial in preventing organ transplant rejection [11], although prolonged HO-1 expression in ischemic and traumatic brain injury lacked a conclusively beneficial effect [12]. Furthermore, a polymorphism in the HO-1 gene promoter region, with longer vs. shorter GT(n) repeats, may be associated with susceptibility to ischemic events [13]. On the other hand, suppression of HO-1 expression was found to be beneficial in brain hemorrhage [14] and a potential therapeutic intervention in Alzheimer's disease [15]. Additionally, HO-1 deficiency in humans results in severe abnormal growth and development [16].

The cytotoxic free radical nitric oxide (NO) plays an important pathogenic role in many neurodegenerative diseases [17-19]. In interleukin (IL)-1β-activated human astrocytes, robust NO production generated by inducible NO synthase (iNOS) has been shown to be either detrimental [20] or beneficial [21] depending on various circumstances. In the presence of the reactive oxygen species (ROS) superoxide (O_2_^-^), NO combines with O_2_^- ^to form the highly toxic radical peroxynitrite (ONOO) which can cause severe damage to neurons. The anti-oxidant defense system present in astrocytes appears to afford a protective effect on surrounding neurons. NO is one among many stimuli that are capable of inducing HO-1 expression [22]. This suggests that the oxidative stress conditions induced by NO can be dampened by the anti-oxidant property of HO-1 to confer an important negative feedback loop.

A few reports have shown that HO-1 induction decreases NO production and iNOS expression, including in a rat model of glomerulonephritis [23], in a human intestinal epithelial cell line [24] and in a lipopolysaccharide (LPS)-induced mouse macrophage cell line RAW264.7 [25]. Increased HO-1 and reduced iNOS expression were also observed in spontaneously hypertensive rats but without a cause-effect relationship being established [26]. As our research laboratory has been interested in the role of glial cells in neuropathogenesis and host defense of the brain in this study we tested the hypothesis that hemin would induce expression of HO-1, which in turn would inhibit iNOS and NO production in human astrocytes stimulated with the pro-inflammatory cytokine IL-1β. Although several signaling pathways are activated by IL-1β in astrocytes, we focused on mitogen activated protein kinases (MAPKs) to determine if the effect of hemin on IL-1β-stimulated astrocytes is mediated through a MAPK signaling pathway. We also looked into the possible effects of hemin on IL-1β-stimulated cytokine and chemokine production to assess whether HO-1 also dampens the production of these inflammatory mediators.

## Methods

### Reagents

The following reagents were purchased from the indicated sources: hemin (ferriprotoporphyrin IX chloride) and Sn (IV) Protoporphyrin IX dichloride (SnPP, 8,13-Bis(vinyl)-3,7,12,17-tetramethyl-21H,23H-porphine-2,18-dipropionic acid tin(IV) dichloride) (Frontier Scientific, Logan, UT); IL-1β, tumor necrosis factor (TNF)-α, CXCL10, Human iNOS Quantikine ELISA Kit, anti-human TNF-α and CXCL10 antibodies (R&D Systems, Minneapolis, MN); anti-p38 and -extracellular signal-regulated kinase 1 and 2 (ERK1/2 or p44/42) MAPK antibodies (Cell Signaling, Beverly, MA); SB203580 (an inhibitor of p38 MAPK) and U0126 (an inhibitor of MAP kinase kinase [MEK]1/2, upstream of ERK1/2) (EMD Biosciences, La Jolla, CA); mouse anti-HO-1 antibody (Assay Designs, Ann Arbor, MI); RNase inhibitor, SuperScript™ III reverse transcriptase and alamarBlue^® ^(Invitrogen, Carlsbad, CA); DNase (Ambion, Austin, TX); oligo (dT)_12-18 _(Promega, Madison, WI); SYBR^® ^Premix Ex Taq™ (Takara, Madison, WI); SYBR^® ^Advantage^® ^qPCR premix (ClonTech, Mountain View, CA); dNTPs (GE Healthcare, Piscataway, NJ); rabbit anti-NOS2 and HO-2 antibodies (Santa Cruz Biotechnology, Santa Cruz, CA); rabbit anti-GFAP (glial fibrillary acidic protein, Dako, Carpinteria, CA); LentiORF™ pLEX-MCS vector (Open Biosystems, Huntsville, AL); Fugene 6 (Roche, Indianapolis, IN); M-PER (Thermo Fisher Scientific, Rockford, IL); Dulbecco's modified Eagle's medium (DMEM), bovine serum albumin (BSA), and 3,3'-diaminobenzidine, 3-(4,5-dimethyl-2-thiazolyl)-2,5-diphenyl-2H-tetrazolium bromide (MTT) (Sigma-Aldrich, St. Louis, MO); acrylamide/bis-acrylamide gel and protein assay (Bio-Rad, Hercules, CA); CDP-Star substrate (Applied Biosystems, Foster City, CA); K-Blue substrate (Neogen, Lexington, KY); heat-inactivated fetal bovine serum (FBS, Hyclone, Logan, UT).

### Preparation of hemin and SnPP

Both hemin and SnPP were dissolved in 0.2 N NaOH, adjusted to physiological pH 7.4 with 1 N HCl, aliquoted in dark brown tubes and frozen at -80°C.

### Astrocyte cultures

Astrocyte**s **were prepared from 16- to 22-week-old aborted human fetal brain tissues obtained under a protocol approved by the Human Subjects Research Committee at our institution. Brain tissues were dissociated and resuspended in DMEM containing penicillin (100 U/ml), streptomycin (100 μg/ml), gentamicin (50 μg/ml) and Fungizone^® ^(250 pg/ml) and plated onto poly-L-lysine (20 μg/ml)-coated 75-cm^2 ^flasks at a density of 80-100 × 10^6 ^cells/flask and incubated at 37°C in a 6% CO2 incubator. Culture medium was changed at a weekly interval. On day 21, flasks were shaken at 180-200 rpm for 16 h followed by trypsinization with 0.25% trypsin in HBSS for 30 min. After adding FBS (final concentration 10%), centrifugation and washing, cells were seeded into new flasks with DMEM followed by medium change after 24 h. The subculture procedure was repeated four times at a weekly interval to achieve highly purified astrocyte cultures (99% of cells reacted with GFAP antibody) which were plated onto 60-mm petri dish (0.6 to 1 × 10^6 ^cells/dish), 6- or 12-well (10^6 ^cells/well) or 48-well (10^5 ^cells/well) plates for protein collection, RNA extraction or ELISA assay.

### Cell culture treatment conditions

Astrocyte culture medium was replaced with DMEM without serum prior to SnPP or hemin treatment. The final serum concentration of 6% was restored at 3 h after the last hemin treatment unless noted. The concentrations of SnPP or hemin used throughout this study did not induce toxicity to astrocyte cultures as verified by MTT, trypan blue dye exclusion and alamarBlue^® ^assays. All experiments containing SnPP or hemin treatment were conducted in the dark with a dim light to minimize inactivation of these compounds. Cell culture plates or petri dishes were kept in a dark box to prevent light exposure.

### Cell viability assay

To determine the effect of hemin or SnPP on astrocyte viability a MTT assay, which provides quantitative assessment of mitochondrial integrity [transformation of tetrazolium salt via mitochondrial succinic dehydrogenases to formazan crystals (purple)], was used. After treatment of astrocytes with hemin or SnPP, MTT (final concentration of 1 mg/ml) was added to cell cultures for 4 h followed by addition of lysis buffer (20% SDS [w/v] in 50% N,N-dimethyl formamide, pH 4.7, adjusted with 2.5% acetic acid and 1 N HCl [32:1]) for 16 h. Cell lysate was collected and absorbance was read at 600 nm (Molecular Devices, Sunnyvale, CA) to reflect possible cytotoxicity caused by treatment. Another cell proliferation and cytotoxicity assay using alamarBlue^®^, in which the living cells convert the nontoxic, cell permeable and non-fluorescent resazurin (blue) to red-fluorescent resorufin (red), was measured at Ex _560 nm _and Em _590 nm _to verify cell viability.

### Enzyme-linked immunoabsorbent assay (ELISA)

After treatment, astrocyte culture supernatants were collected for ELISA measurement [27] of cytokines and chemokines. In brief, 96-well ELISA plate pre-coated with mouse anti-human cytokine/chemokine antibody (2 μg/ml) overnight at 4°C was blocked with 1% BSA in PBS for 1 h at 37°C. After washing with PBS with Tween 20, culture supernatants and a series of dilution of cytokines/chemokines (as standards) were added to wells for 2 h at 37°C. Goat anti-human cytokine/chemokine detection antibody was added for 90 min followed by addition of donkey-anti-goat IgG horseradish peroxidase conjugate (1:10,000) for 45 min. A chromogen substrate K-Blue was added at room temperature for color development which was terminated with 1 M H_2_SO_4_. The plate was read at 450 nm and cytokine/chemokine concentrations were extrapolated from the standard concentration curve.

### NO assay

After treatment, astrocyte culture supernatants were collected to measure nitrite (NO_2_^-^) release using Griess reagent, which reflects NO production in cultures as previously described [20]. In brief, Griess reagent (freshly mixed before use) consisting of equal volumes of 0.1% naphthylenediamine dihydrochloride in distilled H_2_O and 1% sulfanilamide and 6% H_3_PO_4 _in distilled H_2_O, was added in equal volume to astrocyte culture supernatants. After 10 min incubation at room temperature, the mixtures were read with a microplate reader at 550 nm and NO_2_^- ^level was extrapolated from a standard curve generated with a series of concentrations of sodium nitrite (NaNO_2_, 1-62.5 μM). The detection limit for NO_2_^- ^was 0.5 μM.

### iNOS immunoassay

To determine the iNOS concentration in cell lysates, astrocyte cultures (in 12-well plate) were untreated or pretreated with hemin for 24 h prior to IL-1β treatment for 72 h. Cell lysates were collected and assayed according to manufacturer's protocol.

### Immunocytochemical reaction

After treatment, astrocyte cultures plated onto chamber slides were fixed with 4% paraformaldehyde followed by washing with PBS and incubation with 10% normal donkey serum in PBS for 1 h at room temperature (RT). Primary mouse anti-human HO-1 or rabbit anti-GFAP antibodies (1:1000, 1 mg/ml) was added and incubated overnight at 4°C. After washing, secondary antibody (rhodamine- or FITC-conjugate) was added for 1 h at RT followed by viewing under fluorescent microscope.

### pLEX HO-1 vector transfection

Astrocyte cultures were transfected with 1 μg pLEX vector containing either blank or human HO-1 sequences under a cytomegalovirus (CMV) promoter in Fugene 6 reagent for 72 h prior to IL-1β treatment for 72 h. Total cell proteins were collected with M-PER, aliquoted and mixed with 2× sample buffer before being stored at -20°C.

### Real-time polymerase chain reaction

Total RNA extracted from astroyctes after treatment was treated with DNase and reverse transcribed to cDNA with oligo (dT)_12-18_, random hexmer, dNTPs, RNase inhibitor and SuperScript™ III reverse transcriptase. Mixtures of diluted cDNA, primers and SYBR^® ^Premix Ex Taq™ or SYBR^® ^Advantage^® ^qPCR premix were subjected to real-time PCR (Stratagene, La Jolla, CA) according to manufacture's protocol. Primer sequences were sense 5'- TCAACATCCAGCTCTTTGAGGA-3' and antisense 5'-AGTGTAAGGACCCATCGGAGAA-3' for HO-1, sense 5'- TTATGACTCCCAAAAGTTTGACCA-3' and antisense 5'- CCGTCAGTTGGTAGGTTACTGTTG-3' for iNOS, and sense 5'- GACCTGCTGGATTACATCAAAGCACT-3' and antisense 5'- CTTTGGATTATACTGCCTGACCAAGG-3' for HPRT (hypoxanthine phosphoribosyltransferase).

### Western blot

Cell lysates collected after treatment were electrophorezed in 12% (for HO-1, HO-2 or MAPKs) or 7.5% (for iNOS) acrylamide/bis-acrylamide, electrotransfered onto nitrocellulose membrane and probed with antibodies for HO-1, HO-2, iNOS or MAPKs (p38 and p44/42) followed by alkaline phosphatase-conjugated secondary antibodies with chemiluminescence detection using Kodak Image Station (Carestream Health (formerly Kodak), New Heaven, CT).

### Statistical analysis

Data are expressed as mean ± SD or SE as indicated. For comparison of means of multiple groups, analysis of variance (ANOVA) was used, followed by Fisher's PLSD test.

## Results

### Inhibition of iNOS mRNA expression and NO production

**Figure 1:**
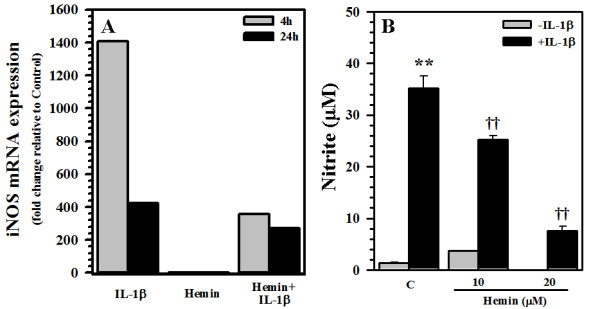
**Inhibition of iNOS expression and NO production in human astrocytes**. Human astrocyte cultures were treated with hemin (10 and 20 μM) for 24 h prior to IL-1β (10 ng/ml) exposure for **A) **4 h or 24 h for RNA extraction to measure iNOS mRNA expression or **B) **72 h to measure NO concentration in culture supernatants. Data presented in A) are representative of 3 separate experiments and in B) are mean ± SE of triplicates from different brain tissue specimens. ** p < 0.01 vs. untreated control (C); ^††^p < 0.01 vs. IL-1β alone.

To test the hypothesis that hemin would inhibit iNOS expression, human astrocyte cultures were pretreated with hemin for 24 h followed by IL-1β treatment for 4 h or 24 h for total RNA isolation. Marked inhibition of iNOS mRNA expression was observed in hemin-pretreated cells (Fig. 1A). In the same hemin treatment paradigm followed by stimulation of astrocytes with IL-1β for 72 h, a similar inhibitory effect of hemin was observed when culture supernatants were assayed for NO (Fig. 1B).

### Hemin-induced HO-1 expression in human astroyctes

**Figure 2:**
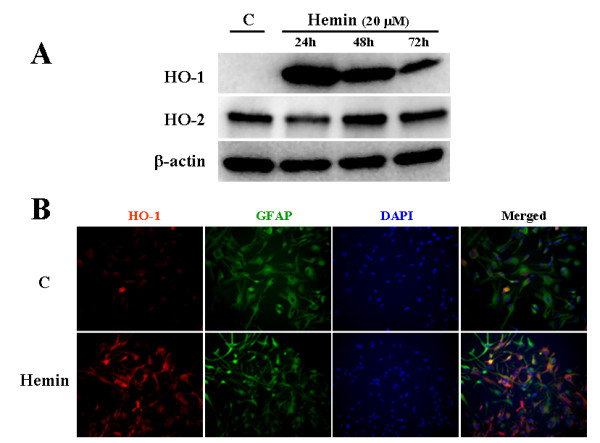
**Induction of HO-1 expression**. **A) **Cell lysates collected from hemin (20 μM)-treated human astrocyte cultures for 24, 48 and 72 h were electrophorezed, transferred to nitrocellulose membrane and probed for HO-1, HO-2 or β-actin (as internal control) expression. C, untreated control. **B) **Immunocytochemical reaction of hemin (20 μM)-treated astrocyte cultures (72 h) for HO-1 (red) or GFAP (green) and counter stained with DAPI (blue) was shown.

To confirm that hemin induces HO-1 in human astrocytes, cells were treated with hemin (20 μM) for 24, 48 and 72 h and HO-1, HO-2 and β-actin (as internal control) expression were assessed by western blot (Fig. 2A). Induction of HO-1 expression by hemin was robust at 24 h and decreased over time, while HO-2 was constitutively expressed in human astrocytes. No effect of hemin on β-actin expression was observed. There was no cytotoxicity induced by hemin measured by MTT (101.95 ± 2.59% of untreated control) or alamarBlue (100.11 ± 3.35% of untreated control) assays. Immunocytochemical reaction also demonstrated no nuclear fragmentation (DAPI staining at 1 μg/ml) in hemin-treated astrocytes indicating no cytotoxicity. It also showed that all astrocytes were GFAP positive and hemin induced robust HO-1 expression, which was co-localized with many, if not all, GFAP positive astrocytes (Fig. 2B).

### Blockade of the inhibitory effect of hemin on NO production

**Figure 3:**
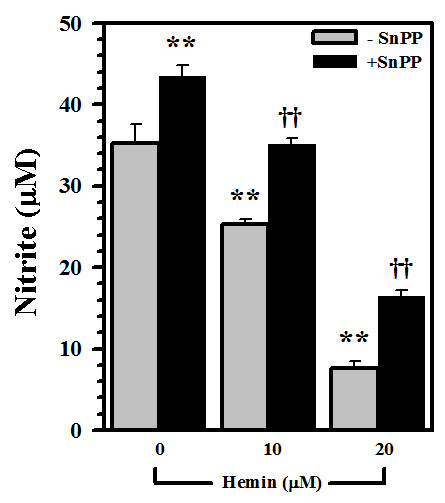
**Blockade of hemin-induced NO inhibition**. Human astrocyte cultures were pretreated with SnPP (10 μM) for 3 h prior to hemin (10 and 20 μM) treatment for 24 h followed by IL-1β (10 ng/ml) exposure for 72 h before measuring NO concentration in culture supernatants. Data presented are mean ± SE of triplicates from 3 separate experiments using different brain tissue specimens. ** p < 0.01 vs. IL-1β alone; ^††^p < 0.01 vs. corresponding hemin+IL-1β.

Pretreatment of human astrocytes with SnPP significantly ameliorated hemin-mediated inhibition of IL-1β-induced NO production, although SnPP did not fully restore the NO level when 20 μM hemin was used (Fig. 3) suggesting involvement of additional mechanism(s). Pretreatment with SnPP appeared to enhance IL-1β-induced NO production suggesting that SnPP itself had no effect on NO production, but rather had exerted inhibition on inducible HO-1 and the constitutive HO-2 [28,29].

### Blockade of the inhibitory effect of hemin on iNOS expression

**Figure 4:**
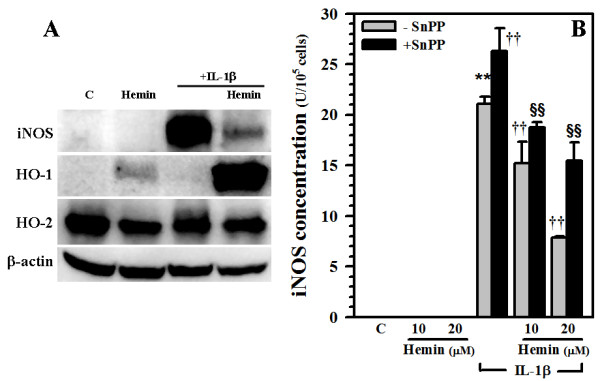
**Blockade of hemin-induced iNOS inhibition**. **A) **Culture lysates (40 μg) collected from human astrocytes treated with hemin (20 μM) for 24 h prior to IL-1β (10 ng/ml) exposure for 72 h were electrophorezed, transblotted to nitrocellulose membrane and probed for iNOS, HO-1, HO-2 or β-actin (as internal control) expression. **B) **Human astrocytes were pretreated with SnPP (10 μM) for 3 h prior to hemin (10 and 20 μM) treatment for 24 h followed by IL-1β (10 ng/ml) exposure for 72 h. Cell lysates were collected, centrifuged and assayed for iNOS expression by ELISA. Sensitivity of iNOS ELISA was 0.05-0.46 U/ml. Data presented are mean ± SE of triplicates from 2 separate experiments using different brain tissue specimens. ** p < 0.01 vs. untreated control (C); ^††^p < 0.01 vs. IL-1β alone; ^§§^p < 0.01 vs. corresponding hemin+IL-β.

Hemin treatment inhibited IL-1β-induced iNOS expression in human astrocytes (Fig. 4A, B). Furthermore, hemin-induced HO-1 expression was further enhanced in the presence of IL-1β (Fig. 4A). The constitutively expressed HO-2 was minimally changed by hemin and IL-1β treatment while no effect on β-actin expression was found (Fig. 4A). Pretreatment with the HO-1 inhibitor SnPP significantly reversed the inhibitory effects of hemin on IL-1β-induced iNOS expression (Fig. 4B). As mentioned above, SnPP also enhanced IL-1β-induced iNOS indicating that SnPP not only inhibited HO-1, but also may have relieved the inhibitory effect of endogenous components, e.g, HO-2, exerted upon iNOS.

### Overexpression of HO-1 inhibits iNOS expression

**Figure 5:**
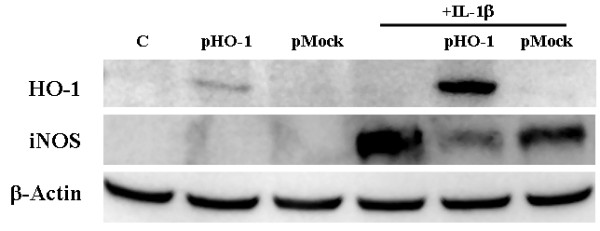
**Effects of HO-1 overexpression**. Human astrocyte cultures were transfected with 1 μg pLEX expression vector containing blank (pMock) or human HO-1 (pHO-1) sequences under a CMV promoter in Fugene 6 reagent for 72 h prior to IL-1β exposure for 72 h followed by protein collection with M-PER. Equal amount of proteins were loaded onto 12% (for HO-1) or 7.5% (for iNOS) acrylamide/bis gel for electrophoresis, transblotted onto nitrocellulose membrane and probed with mouse anti-HO-1 or rabbit anti-iNOS or anti-β-actin (as internal control) antibodies. C; untreated control.

To further investigate the role of HO-1 in iNOS expression, human astroyctes were transfected with a pLEX expression vector containing human HO-1 sequences under a CMV promoter for 72 h. The transfection efficiency was approximately 30% in human primary astrocytes and this treatment was associated with expression of HO-1, while treatment with a blank sequence-containing vector was not (Fig. 5). In combination with IL-1β, HO-1 expression was further enhanced (Fig. 5). IL-1β-induced iNOS expression was markedly downregulated by overexpression of HO-1, further demonstrating the inhibitory effect of HO-1 on iNOS expression (Fig. 5). No effect on β-actin expression was found.

### Immunocytochemical reaction of IL-1β-induced HO-1 expression

**Figure 6:**
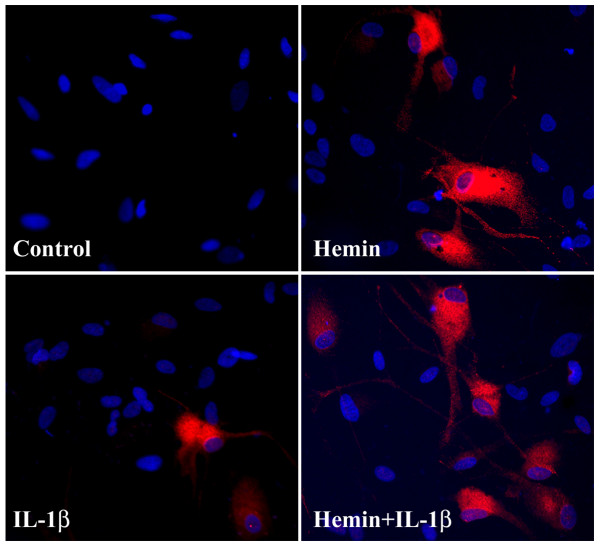
**Immunocytochemical reaction of IL-1β-induced HO-1 expression**. Human astrocyte cultures were pretreated with hemin for 24 h prior to IL-1β exposure for 72 h followed by fixation and immunocytochemical reaction for HO-1. Untreated control (C), blue - DAPI nuclei staining and red - HO-1 reaction.

Although IL-1β treatment induced undetectable HO-1 expression by western blot, induction of HO-1 was detectable by immunocytochemical reaction (Fig. 6), possibly due to different detection sensitivities between these methods.

### Involvement of p38 MAPK

**Figure 7:**
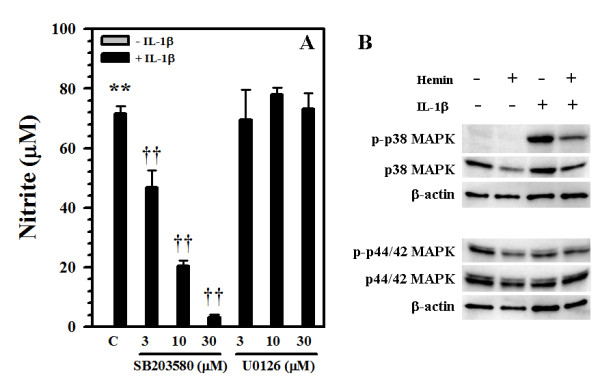
**Involvement of p38 MAP kinase**. **A) **Human astrocytes were pretreated for 1 h with the p38 MAPK inhibitor SB203580 or MEK1/2 inhibitor U0126 (3, 10, 30 μM) prior to IL-1β (10 ng/ml) exposure for 72 h. Culture supernatants were collected to measure NO levels by Griess reagent. Data presented are mean ± SE of triplicates of 2-3 separate experiments using astrocyte cultures from different brain tissue specimens. **p < 0.01 vs. untreated control (C); ^††^p < 0.01 vs. IL-1β alone. **B) **After replacing culture media with DMEM without serum, human astrocytes were treated with hemin (20 μM) for 24 h prior to IL-1β (10 ng/ml) exposure for 30 min. Cell lysates were electrophorezed, transblotted to nitrocellulose membrane and probed for p38 or p44/42 MAPK or β-actin (as internal control). Data presented are representative of 3 separate experiments using different brain tissue specimens.

Because IL-1β is known to trigger activation of both p38 and ERK1/2 (p44/42) MAPK signaling pathways in human astrocytes [30], we studied the effects of specific inhibitors of p38 (SB203580) and ERK1/2 (p44/42) (U0126) MAPK on NO production. As shown in Fig. 7A, we found that NO production was dependent on p38 but not p44/42 MAPK activation. The inactive inhibitor of p38 MAPK (SB202474) had no effect on NO production (data not shown). Treatment with these inhibitors alone (0.3-30 μM) did not induce astrocyte toxicity by MTT or alamarBlue assay (data not shown). Because hemin treatment inhibited IL-1β-induced NO production, we investigated the effect of hemin on IL-1β-induced p38 MAPK activation. Hemin alone minimally activated MAPK, however, it markedly down-regulated IL-1β-induced p38 but not p42 MAPK activation (Fig. 7B), suggesting the involvement of p38 MAPK in the inhibitory effects of hemin on NO production. No effect on β-actin expression was found (Fig. 7B).

### Inhibition of cytokine/chemokine production

**Figure 8:**
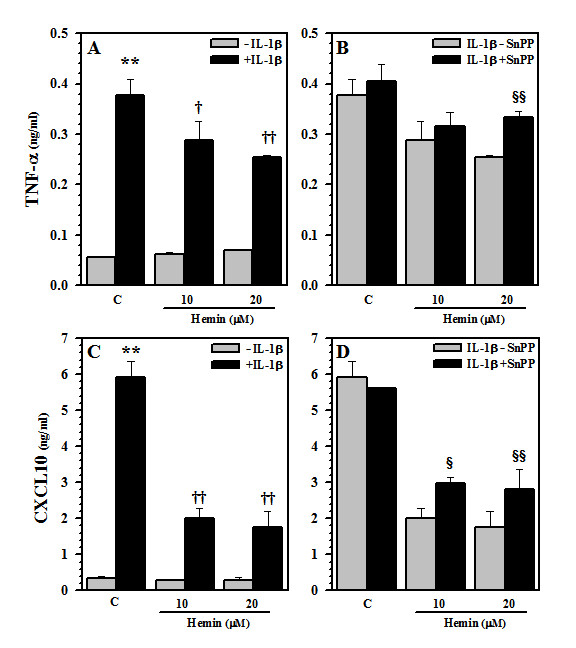
**Regulation of TNF-α and CXCL10 production**. Culture supernatants collected from human astrocytes pretreated with (B, D) or without (A, C) SnPP (10 μM) for 3 h prior to hemin (10 and 20 μM) treatment for 24 h followed by IL-1β exposure were measured for **A, B) **TNF-α (24 h) or **C, D**) CXCL10 (48 h) by ELISA. Data presented are mean ± SE of triplicates from 2 separate experiments using different brain tissue specimens. ** p < 0.01 vs. untreated control (C); ^†^p < 0.05 and ^††^p < 0.01 vs. IL-1β alone; ^§^p < 0.05 and ^§§^p < 0.01 vs. corresponding hemin+IL-1β.

After establishing the inhibitory effect of hemin on iNOS expression and NO production, we investigated whether hemin also would suppress the production of other inflammatory mediators, i.e. cytokines and chemokines, produced by IL-1β-stimulated human astrocytes. Previously, we found that IL-1β-activated human astrocytes release TNF-α and CXCL10 [27]. Following hemin pretreatment, IL-1β- induced TNF-α and CXCL10 production was down-regulated (Fig. 8A, C) and this inhibition was blocked significantly by SnPP (Fig. 8B, D) suggesting the involvement of HO.

## Discussion

In the current study, we demonstrated that hemin robustly induces HO-1 expression in human astrocytes and that pretreatment with hemin significantly inhibited IL-1β-induced iNOS expression, NO production, and TNF-α as well as CXCL10 release. Furthermore, we showed that this inhibitory effect was markedly reversed by the HO activity inhibitor tin-protoporphyrin (SnPP), suggesting the involvement of an HO-mediated mechanism. IL-1β-induced NO production is known to be p38 MAPK-dependent, and we found that hemin treatment down-regulated IL-1β-induced p38 MAPK, suggesting the involvement of this intracellular signaling pathway in hemin's inhibitory action. Interleukin-1β activates astrocytes robustly to produce inflammatory mediators including cytokines, chemokines, and NO [27,31,32], which may contribute to autocrine and paracrine effects on neighboring neuronal and glial cells. Nitric oxide is one of the stimuli known to induce HO-1 [33], which exerts a possible feedback inhibition on NO, as seen in this study.

The role of HO-1 under different experimental paradigms and disease conditions has been found to be either beneficial or damaging and its protective function is debatable. Due to inflammatory mediator production by IL-1β-activated astrocytes leading to potential harmful consequences, our hypothesis was that hemin induction of anti-inflammatory HO-1 expression in IL-1β-activated astrocytes would be beneficial. The results of this study support the notion that hemin inhibits IL-1β-induced iNOS expression and NO production in human astrocytes and are in agreement with findings of others using cell types not found within the nervous system [23-25]. The interplay and negative feedback interaction between HO-1 and iNOS that we found in this study has been observed in the study of glomerulonephritis [23]. This phenomenon has also been suggested to involve a reduction of the available heme pool [34] for de novo iNOS synthesis, CO interacting with iNOS heme moiety [35] and iron down-regulation of iNOS transcription [36].

In this study we also confirmed the finding that NO production is dependent on p38 MAPK [37]. The down-regulation of p38 MAPK by hemin pretreatment suggests involvement of the p38 MAPK signaling pathway in the inhibitory effect of hemin on IL-1β-induced NO production. In the murine macrophage cell line RAW264.7, hemin was found to attenuate LPS-induced NF-κB activation [38]. Silencing HO-1 was found to enhance LPS-induced nuclear factor (NF)-κB activation suggesting an inhibitory role of HO-1 on NF-κB activation [39] which is also required downstream for NO production [40]. Thus, hemin could also inhibit IL-1β-stimulated downstream NF-κB activation in astrocytes. Our demonstration that SnPP blocks hemin-suppressed IL-1β-induced inflammatory TNF-α and CXCL10 production in human astrocytes corresponds well with the finding that overexpression of HO-1 inhibited LPS-induced TNF and IL-1β expression in THP-1 cells [39], providing further evidence for the anti-inflammatory effect of HO-1.

Several caveats and limitations in our study must be acknowledged. The constitutive expression of HO-2 in our human primary astrocytes may also have contributed to the inhibition of NO [41] as shown by non-selective SnPP treatment on IL-1β. Another possible explanation is that SnPP alters an unknown mechanism leading to the enhancement of IL-1β-induced iNOS expression and NO production in astrocytes. Although there was no cytotoxicity detected by either MTT or alamarBlue assays, we observed that hemin treatment altered astrocyte morphology to a smaller cell size without changing β-actin expression. We also observed minor inhibition of GFAP expression by hemin. Hemin-induced HO-1 expression was observed in about 50% of astrocytes; this could be due to subtypes of and/or delayed response among astrocytes in cultures. Transfection of astrocytes with an HO-1 expression vector demonstrated the inhibitory effect of HO-1 on iNOS, but potential mechanisms involving byproducts from the HO reaction, i.e., CO, iron, biliverdin and bilirubin, should not be ignored.

In conclusion, we have demonstrated *in vitro *the robust induction of HO-1 expression in human astrocytes exposed to hemin. Induced HO-1 expression exerts an inhibitory effect on iNOS expression and NO production in IL-1β-stimulated human astrocytes and the inhibitory effects of hemin are mediated mainly through HO-1 induction and associated with reduced activation of p38 MAPK. Extrapolation of these *in vitro *human brain cell culture results to *in vivo *models should be undertaken with caution as there are species and response differences to be expected. However, these findings support the concept that HO-1 expression in astrocytes is an antioxidant defense system in the face of neuroinflammation.

## Competing interests

The authors declare that they have no competing interests.

## Authors' contributions

WSS carried out half of experiments, participated in the design, statistical analysis and wrote the initial version of the manuscript. SH carried out another half of experiments and participated in the design. ARN made the construct of pLEX HO-1 expression vector. JRL contributed to the design. GMV contributed expertise in HO-1 and experimentation using hemin. RBR conceived the study and was responsible for editing and revising the final version of the manuscript. All authors have read and approved the final version of the manuscript.
